# Role of the Gut Microbiota in the Pathophysiology of Autism Spectrum Disorder: Clinical and Preclinical Evidence

**DOI:** 10.3390/microorganisms8091369

**Published:** 2020-09-07

**Authors:** Léa Roussin, Naika Prince, Paula Perez-Pardo, Aletta D. Kraneveld, Sylvie Rabot, Laurent Naudon

**Affiliations:** 1 Université Paris-Saclay, INRAE, AgroParisTech, Micalis Institute, 78350 Jouy-en-Josas, France; sylvie.rabot@inrae.fr; 2 Division of Pharmacology, Utrecht Institute for Pharmaceutical Sciences, Faculty of Science, Utrecht University, Universiteitsweg 99, 3584 CG Utrecht, The Netherlands; n.z.prince@uu.nl (N.P.); p.perezpardo@uu.nl (P.P.-P.); a.d.kraneveld@uu.nl (A.D.K.); 3 Université Paris-Saclay, INRAE, AgroParisTech, CNRS, Micalis Institute, 78350 Jouy-en-Josas, France; laurent.naudon@inrae.fr

**Keywords:** autism spectrum disorder, microbiota-gut-brain axis, immune system, tryptophan metabolism, animal models of autism spectrum disorder

## Abstract

Autism spectrum disorder (ASD) is a neurodevelopmental disorder affecting 1 in 160 people in the world. Although there is a strong genetic heritability to ASD, it is now accepted that environmental factors can play a role in its onset. As the prevalence of gastrointestinal (GI) symptoms is four-times higher in ASD patients, the potential implication of the gut microbiota in this disorder is being increasingly studied. A disturbed microbiota composition has been demonstrated in ASD patients, accompanied by altered production of bacterial metabolites. Clinical studies as well as preclinical studies conducted in rodents have started to investigate the physiological functions that gut microbiota might disturb and thus underlie the pathophysiology of ASD. The first data support an involvement of the immune system and tryptophan metabolism, both in the gut and central nervous system. In addition, a few clinical studies and a larger number of preclinical studies found that modulation of the microbiota through antibiotic and probiotic treatments, or fecal microbiota transplantation, could improve behavior. Although the understanding of the role of the gut microbiota in the physiopathology of ASD is only in its early stages, the data gathered in this review highlight that this role should be taken in consideration.

## 1. Introduction

Autism spectrum disorder (ASD) is one of the most prevalent neurodevelopmental disorders, characterized by impairment in social behavior, communication, prevalence of repetitive and stereotyped behavior and lack of adaptation to change. It can sometimes also involve cognitive impairments and anxiety disorders. Although the behavioral diagnosis of ASD has improved in the past decade, it is still very hard to characterize, especially for high-functioning ASD. In addition, the diagnosis is only possible after 18 months of age and cannot be confirmed until a later age, which compromises any preventive measures [1,2].

In the last 50 years, the prevalence of ASD has tremendously increased, more than 35-fold since the 1970s, and is now estimated at 1 in 160 children worldwide, 1 in 54 in the United States and 1 in 89 in the European Union [3,4,5].

In the early 1990s, ASD was believed to be due at 90% to genetic factors and highly heritable due to the high risk for siblings of ASD patients. More recent studies described a genetic heritability of around 50%. However, these data are difficult to assess accurately, as the genetic variants responsible for ASD are also associated with other neurodevelopmental disorders. The same studies have proven that environmental factors also play an important role in this disorder and can in part explain such an increase in the prevalence of ASD [6,7,8,9,10]. 

It is important to point out that the prevalence of gastrointestinal (GI) symptoms is four-times higher in ASD children than in typically developing (TD) children and that many studies report a specific GI phenotype in ASD, characterized by increased gut permeability and abnormal immune function in the gut [11]. Interestingly, a recent study reports that ASD children with functional GI disorders show a distinct gut microbiota and immune signature compared to TD children with the same GI symptoms. This study also found a correlation between the ASD-specific dysbiosis (more specifically the increase in species from the *Clostridiales* order) and GI symptoms (inflammation and abdominal pain) [12]. This suggests that these GI symptoms are an integral part of ASD pathophysiology, and are in interaction with the gut microbiota and the immune system.

The involvement of the microbiota-gut-brain axis in ASD has been the focus of numerous studies over the past decade. The purpose of this review is to summarize key findings from clinical and preclinical studies and to describe how ASD-related symptoms can be affected by the gut microbiota. This review begins by describing the changes in microbiota composition and activity that have been first observed in ASD patients but have then also been found in various murine models of ASD over the past decade. It then documents the clinical and preclinical evidence of the implication of the gut microbiota and its metabolites in some important ASD biomarkers such as inflammation and immune impairments, as well as alterations in tryptophan (Trp). Finally, the second part of this review examines the effect of intervention studies targeting the gut microbiota on behavior in ASD patients and animal models of ASD. 

## 2. Clinical and Preclinical Evidence for Involvement of the Gut Microbiota in Various Aspects of ASD

### 2.1. Dysbiosis and Changes in Bacterial Metabolites in ASD

#### 2.1.1. Clinical Evidence

In 2000, Sandler et al. [13] hypothesized that dysbiosis due to antibiotic treatment in young children was involved in the apparition of regressive autism observed in some of those children. They postulated that this was due to colonization of neurotoxin-producing bacteria, and started a clinical trial on 18 children with a vancomycin treatment with the aim of eliminating these bacteria. They observed improvement in the behavior of those children during the treatment, but it did not last after stopping the treatment. Although the association between early-life antibiotic treatment and ASD has not been confirmed since then [14], this princeps study proved the existence of a causative link between modification of the gut microbiota composition and behavior, in a subset of children with ASD. In 2012, the same team published a summary of their research on the subject, highlighting a dysbiosis in ASD children. One genus, *Desulfovibrio* was present in 50% of ASD children, some of their siblings, but never in unrelated controls. The proportion of *Desulfovibrio* correlated with severity of ASD symptoms [15].

Since then, many research teams have been investigating the gut microbiota of ASD children, and most of them have observed a dysbiosis. Recently, two meta-analyses compared these data in an attempt to identify specific genera or species with a consistent pattern of change across studies. The first one [16] analyzed 9 papers and the second [17] studied 18 papers including 8 from the 9 papers reviewed in the first meta-analysis. Both meta-analyses reported a decrease in *Bifidobacterium* and increase in *Faecalibacterium* and *Clostridium* in ASD children despite high interstudy heterogeneity. Only Iglesias-Vasquez et al. (2020) [17] reported differences at the phylum level, such as higher *Bacteroidetes/Firmicutes* ratio in ASD children, or elevated relative abundance of *Proteobacteria*. The two meta-analyses present discrepancies, as Xu et al. (2019) [16] reported a decrease of *Bacteroides* and *Parabacteroides* in ASD children when Iglesias-Vázquez et al. (2020) [17] reported an increase of both those genera. Furthermore, Xu et al. (2020) [16] observed a lower abundance of *Akkermansia* in ASD children when Iglesias-Vásquez et al. (2019) [17] reported no difference. Around the same time, two systematic reviews were also published by Ho et al. (2020) [18] and Bezawada et al. (2020) [19] who compared 26 and 28 studies, respectively, including 14 and 16 of the studies included in any of the meta-analyses. Both underlined the heterogeneity of results among studies and reported a few consistent results. They pointed out that many studies observed an increase in some *Clostridium* species, and a lower proportion of *Bifidobacterium*. Bezawada et al. (2020) [19] also reported that the *Sutterella* genus was found to be more abundant in ASD children in many studies.

Overall, those meta-analyses and reviews confirm the presence of dysbiosis in ASD, despite heterogeneous results among studies. These could be due to methodological differences, but also to the fact that the different cohorts come from multiple countries with different lifestyles and dietary habits. In addition, the age groups of the children recruited in the different studies vary significantly, with some including children as young as 2 years old, an age at which the gut microbiota is not completely stabilized [20]. Despite this, there seems to be a rather consistent increase of *Clostridium*, which is considered to be a putative harmful genus, and a decrease in *Bifidobacterium* which is considered beneficial. Surprisingly, however, both meta-analyses report an increase in *Faecalibacterium* in ASD patients, when the only known species from this genus, *Faecalibacterium prausnitzii,* is considered a beneficial bacterium with anti-inflammatory properties [21].

It is interesting to point out that among the different studies; the control groups differed in their constitution. They were of three types, either composed only of siblings of ASD children, or only of unrelated individuals or of both siblings and unrelated individuals. In the studies with both types of control groups, the sibling group seemed to have a different microbiota profile compared to unrelated individuals, and was sometimes closer to the “ASD profile” [22,23,24,25,26]. This is not surprising considering the influence of genetics and environment on gut microbiota composition. Recent unpublished work from Luna et al. presented at the 74th Annual Meeting Society of Biological Psychiatry Chicago (May 2019) [27] reported a significant difference between the microbiota of TD siblings of ASD children and unrelated controls. This team also reported an influence of GI symptoms on the gut microbiota composition. This heterogeneity in the composition of the ASD and control groups might explain in part the variability between studies. Only an increase in the number of studies and a better standardization of the composition of groups could address this issue. 

The existence of a dysbiosis in the gut of many ASD children is now well-accepted but its precise nature is still not completely understood. In order to understand the impact of this dysbiosis on health, researchers have been focusing on bacterial metabolites that are differentially modulated in ASD children. Different teams have found an increase in urinary p-cresol, a bacterial metabolite derived from tyrosine, in young children with ASD [28,29,30,31]. These teams hypothesized that this increase could be due to a higher level of p-cresol producing bacteria such as *Clostridium difficile*. However, a study by Gabriele et al. (2016) [32] reported that levels of p-cresol did not correlate with elevated proportions of *Clostridium* species, but correlated with slow intestinal transit. Although the increase of urinary p-cresol in young children with ASD has been observed several times, there is little evidence so far explaining the mechanisms underlying this increase. 

Short chain fatty acids (SCFAs) are considered to be key actors of the microbiota-gut-brain axis, and their involvement in multiple neurological disorders has been increasingly described [33]. In the context of ASD, some studies have reported altered fecal levels of SCFAs in ASD children, but with great diversity in results [34,35,36]. Adams et al. (2011) [34] reported a decrease in total SCFAs in stool of ASD patients while Wang et al. (2012) [35] reported an increase. In Liu et al. (2019) [36] acetate and butyrate levels were decreased in stools of ASD patients while valerate levels were increased. Only Wang et al. (2012) [35] reported significant alteration in propionate levels, which they found to be elevated in stools of ASD patients. Finally, Averina et al. (2020) [37] found decreased expression of genes related to production of butyrate in the metagenome of ASD children. 

As of now, there is still little clinical evidence of the impact of those SCFAs in ASD pathophysiology, most of the evidence comes from animal studies and will be discussed later in this review. Interestingly, patients suffering from propionic acidosis, a genetic disorder characterized by an accumulation of propionate, present neurodevelopmental delay, and a recent publication reported a very high prevalence of ASD (21%) in patients with this disease [38]. Propionate can increase oxidative stress, thus influencing mitochondrial activity. Mitochondrial dysfunction has been reported in many ASD patients and believed to play a role in its pathophysiology [39].

Frye et al. (2016) [40] showed that lymphoblastoid cell lines (LCL) derived from ASD patients had a different response to propionate than LCL from control subjects, especially in an oxidative environment, where propionate induced an overproduction of ATP and mitochondrial dysfunction. In another similar in vitro study, Rose et al. (2018) [41] found that LCL derived from ASD patients also responded differently to butyrate than LCL from control subjects. In LCL from controls, butyrate decreased mitochondrial respiration when it did not significantly alter it in LCL from ASD patients and increased it in LCL from ASD patients with mitochondrial dysfunction.

#### 2.1.2. Preclinical Evidence

Because of the multifactorial aspect of ASD, a number of murine models have been developed. Some are genetic models, like the Shank3 KO, NL3^R451C^ or PCDH9 KO models, based on extinction or mutation of genes known to be involved in some cases of ASD [42,43,44,45], or the BTBR mouse strain, considered an idiopathic ASD model, based on its behavioral phenotype [46]. There are also many environmental models either based on a challenge during gestation (maternal high-fat diet (MHFD), maternal immune activation (MIA), maternal exposure to valproic acid (VPA)) or during life (cow’s milk allergy (CMA)) [47,48,49,50]. All those ASD models have been classified as such based on the fact that they present altered behaviors related to ASD symptoms (social interaction and communication deficits, stereotyped behaviors). However, a growing number of studies report GI symptoms in some of these models similar to those observed in ASD patients. More precisely, increased intestinal permeability was found in Shank3 KO, BTBR and MIA mice [51,52,53,54]. Abnormal cytokine profiles have been found in the gut of BTBR, MIA and MHFD mice [52,54,55] and an increase of myeloperoxidase (MPO) expression (marker of inflammation) was found in the ileum of VPA mice [56]. Finally, Hosie et al. (2018) [57] reported a faster transit associated with an increase of inhibitory signaling in the GI epithelium in NL3^R451C^ mice, when the opposite was observed in BTBR mice [53]. Interestingly, many studies have also found dysbiosis in those models, detailed in Table A1 and Table A2 (Appendix A). Although the nature of the dysbiosis is very different among the different models, there are a few similarities. Firstly, a decrease in α-diversity has been described in Shank3 KO, BTBR, MIA and MHFD mice and VPA rats [53,55,58,59,60,61]. However, other studies in Shank3B KO, NL3^R451C^, BTBR, MIA and VPA mice did not observe any change in α-diversity [52,54,57,62,63]. All of the studies included in Table A1 and Table A2 that assessed β-diversity observed a difference between controls and model animals, except for Hsiao et al. (2013) [54]. At the phylum level, an increase in *Bacteroidetes* and a decrease in *Firmicutes* was observed in BTBR and MIA mice, and in male VPA rats [53,59,60,61]. This is in agreement with the elevated *Bacteroidetes/Firmicutes* ratio in ASD patients described in a meta-analysis previously mentioned [17]. However, other studies reported an increase of *Firmicutes* in Shank3 KO, VPA and MHFD mice [51,55,63,64] as well as a decrease in *Bacteroidetes* in VPA and MHFD mice [55,63]. The increase in *Proteobacteria* reported in ASD patients was not seen in ASD mice models except in Shank3 KO mice by Sauer et al. (2019) [51]. Plus, a decrease in this phylum was observed in BTBR mice by Coretti et al. (2017) [52]. At lower taxonomic levels, as in ASD patients, a decrease in *Lactobacillus* has been observed in MIA and Shank3 KO mice [54,58] as well as a decrease in *L. reuteri* in Shank3 KO, Shank3B^-/-^ and BTBR mice [58,62] and a decrease in *L. brevis* and *L. ruminis* in Shank3 KO mice [58]. However, Coretti et al. (2017) [52] found increased *Lactobacillus* in male BTBR mice. The *Prevotella* genus, which has been found to be decreased in ASD patients by Kang et al. (2013) [65], was also decreased in Shank3 KO mice [58] but was increased in MIA and BTBR mice [52,59,60]. Although changes in proportion of *Clostridium* species seem to be recurrent in ASD patients, they were only observed in BTBR mice by Newell et al. (2016) [59], who found decreased and increased levels of various *Clostridium* species in cecal contents and feces, respectively. This study underlies important differences between cecal and fecal composition, which has to be taken in consideration, as most of the studies cited only assessed microbial composition of feces. Plus, most studies used only male mice; however, among the few that used both male and female mice, most observed strong sex-related differences in microbiota composition [52,58,61,63]. Both of those criteria should be considered in future studies.

Overall, the bacterial alterations observed in ASD models vary considerably between studies and models, and do not necessarily reflect the changes observed in ASD patients. However, the occurrence of those alterations in multiple genetic and environmental rodent models of ASD is a strong indicator of the implication of the microbiota-gut-brain axis in ASD pathophysiology. Another observation reinforces this assumption: germ-free (GF) mice, which are devoid of microbiota, present some altered behaviors related to ASD, such as reduced social interaction, and increased stereotyped behavior [49,66] and thus have been proposed as an environmental ASD model. 

Interestingly, there are also reports of altered bacterial metabolites levels in different ASD models, similar to what is observed in ASD. In the MIA model, alterations in several serum metabolites have been observed, in particular, 4-ethylphenylsulfate (4-EPS), a metabolite that is derived from the bacterial metabolite 4-ethylphenol, was found to be drastically increased. 4-EPS is derived from tyrosine, and is structurally close to p-cresol. Interestingly, a probiotic treatment with *B. fragilis* NCTC 9343 restored normal serum levels of 4-EPS and ameliorated anxiety-like behavior, but neither social nor repetitive behaviors in the MIA model [54]. A whole range of intestinal bacteria such as *Coriobacteriaceae, Enterobacteriaceae, Fusobacteriaceae* and *Clostridium* clusters I and XIVa, can catabolize tyrosine into aromatic derivatives, including p-cresol [67]. However, the reasons behind such an increase in this model are currently not known. Interestingly, in a recent report from Bermudez-Martin et al. (2020) [68] a 4-week administration of p-cresol in the drinking water changed microbiota composition and induced impaired social behavior and increased repetitive behavior in wild-type (WT) mice. P-cresol treatment also impaired excitability of dopaminergic neurons in the ventral tegmental area of those mice, a circuit involved in the social reward system [69]. Plus, the authors showed that the effect of p-cresol on behavior was dependent on microbiota composition, as fecal microbiota transplantation (FMT) from p-cresol treated mice to WT mice induced the similar behavioral impairments, and, in contrast, FMT from WT mice to p-cresol-treated mice restored normal social behaviors [68].

The implication of SCFAs has also been investigated in ASD models. In the BTBR model, Golubeva et al. (2017) [53] reports decreased levels of acetate and isobutyrate, but increased levels of butyrate. In addition, increased levels of butyrate have been observed in male VPA mice [54]. Neither of those studies reported on a difference in the levels of propionate. However, there has been reports of an effect of propionate on ASD-related behavior, as its administration to rats alters social behavior, increases repetitive behaviors and alters cognitive functions [70,71,72]. In addition, similarly to what is observed in the VPA model, mother exposure to propionate induces social deficits in the offspring [73]. On the contrary, butyrate improves social deficits in the BTBR mouse model [74], which is surprising considering that butyrate levels are increased in this model [53].

### 2.2. Influence of the Gut Microbiota on Immune System Dysregulation in ASD 

#### 2.2.1. Clinical Evidence

Immune system impairments such as higher blood levels of pro-inflammatory cytokines, dysfunctional immune cells or presence of antibodies targeting brain proteins, have been observed in many ASD children, and in their mothers during pregnancy and post-partum. Interestingly, studies have showed that those increases in pro-inflammatory cytokines can correlate with the severity of some behavioral symptoms [75,76,77]. 

Many clinical studies report higher prevalence of ASD following bacterial or viral infection during pregnancy, which could lead to an inflammatory environment in the placenta and amniotic fluid. It has been hypothesized that those infections, whether they occur before or after birth, could play a crucial role in ASD pathogenesis, as they can influence important neurodevelopmental mechanisms, like microglial maturation and synaptic pruning [77].

The gut microbiota and the immune system are intrinsically linked. It is accepted that a major constitutive function of the immune system is to control the microbiota and reinforce the intestinal barrier. In turn, the microbiota also has a direct effect on the immune system, as bacterial metabolites or compounds can influence differentiation of immune cells, or regulate their activity, not only in early postnatal development but throughout the lifespan. The mammalian immune system has co-evolved with the establishment of the microbiota, to reach a symbiotic relationship. However, this relationship can become more deleterious depending on genetic background, environmental challenges or changes in nutrition [78,79].

Because of those observations, it has been hypothesized that one way of action of the gut microbiota in ASD was through its action on the immune system, more specifically on the balance between T regulatory cells (Treg) and effector T cells, such as T helper (Th) cells in the gut. Those Th cells are the results of naïve CD4+ T cells differentiation. One subtype of Th cells, Th17, is pro-inflammatory and can be involved in autoimmunity. On the contrary, Treg cells are anti-inflammatory and play a protective role against autoimmunity. Disruption of Treg/Th17 balance has been linked to the pathophysiology of many autoimmune diseases, and could also be involved in ASD [80,81]. Although differentiation is mostly driven by immune signals such as chemokines or cytokines, the Treg/Th17 balance could be influenced by an altered microbiota. Indeed, differentiation into Treg can be induced by some species of *Clostridiales* and by *Bacteroides fragilis*, whereas the differentiation into Th17 cell can be induced by some segmented filamentous bacteria (SFB) [82].

Interestingly, in Rose et al. (2018) [83] pro-inflammatory cytokines were elevated in the serum and gut of ASD patients, and this elevation was higher in children with ASD and GI symptoms than in children with ASD and no GI symptoms. In addition, a more recent study by the same team characterized circulating effector T cell populations in ASD patients with or without GI symptoms in comparison to TD controls. They found increased levels of IL-17 positive CD4+ and CD8+ T cells in ASD patients compared to controls, and this increase was even stronger in ASD patients with GI symptoms. The levels of IFNγ were also increased in ASD patients with GI symptoms compared to ASD patients without. Furthermore, they found decreased regulatory T cells in both ASD groups compared to TD, and a decrease in Treg/Th17 ratio in ASD patients with GI symptoms [84]. These observations have been completed by the fact that some of the bacterial species altered in ASD patients appear to be associated with overproduction of interferons (IFN) and pro-inflammatory cytokines in the gut. Indeed, a correlation was found between fecal levels of *Faecalibacterium* and increased levels of genes involved in type I IFN and IFN-γ signaling in immune cells of ASD children compared to TD-unrelated controls [85]. Type I IFN signaling induces antimicrobial programs and is involved in regulation of innate and adaptive immunity, but also in autoimmune diseases [86]. In addition, Luna et al. (2017) [12] reported the existence of a correlation between levels of multiple bacterial species in children with ASD and GI symptoms, and elevated levels of various cytokines in their blood.

Clinical studies also report a neuroinflammatory state in ASD, characterized by proliferation and morphological modification of microglia and astrocytes into a reactive state in the brain. Indeed, post mortem observation of the brains of ASD patients revealed increased glial fibrillary acidic protein (GFAP)-positive cells and GFAP protein levels (marker of astrogliosis). Plus, increased markers of reactive microglia and astrocytes were found in various brain regions, but most notably in the cerebellum. Similarly to what has been observed in the blood of ASD patients; increased levels of many pro-inflammatory cytokines were found in the brain and cerebrospinal fluid of ASD patients post mortem [87,88]. Suzuki et al. (2013) [89] used positron emission tomography (PET) and observed more reactive microglia in various brain regions of ASD patients compared to controls, most strikingly in the cerebellum. Those neuroglial alterations are believed to play a role in ASD pathophysiology, as microglia and astrocytes are involved in neurodevelopment, in part via synaptic pruning. In physiological conditions, synaptic pruning consists in reinforcement of important connections and removal of redundant connections by phagocytosis. This process plays a crucial role in wiring the brain during development and is involved in plasticity during life, but could be deleterious if overly activated. Thus, a reactive state of microglia and astrocytes in development and throughout life in ASD may result in changes in neuronal morphology and connectivity which could contribute to behavioral and cognitive alterations [90]. To our knowledge there are no clinical studies that link those neuroinflammatory defects to the impaired microbiota in ASD. Only animal studies, as described below, provide evidence for a crucial role of a complex microbiota in microglial maturation and function.

#### 2.2.2. Preclinical Evidence

First, in studies in the VPA-induced murine model of ASD, markers for neuroinflammation were found to be increased in the dorsal hippocampus associated with marked changes in microbiota composition in the intestinal tract [56,63]. In addition, a study by Erny et al. (2015) [91] showed that absence of microbiota from birth (GF mice) or depletion during life (SPF mice treated with antibiotics) led to immature microglia exhibiting a blunted response to LPS challenge. This was reversed by co-housing with SPF mice with a complex microbiota. Interestingly, a normal microglial phenotype was also restored by the administration of a cocktail of SCFAs in the drinking water. Finally, another more recent study demonstrated therapeutic effects of a Trp-derived bacterial metabolite, Indoxyl-3-sulfate (I3S), on microglia- and astrocyte-related neuroinflammation in a mouse model of multiple sclerosis [92]. Those results proved that a complex microbiota and its metabolites are necessary for microglia maturation and influence microglia and astrocyte function both during development and throughout life. 

Animal studies also provide most of the evidence on the implication of the microbiota in the other immune alterations observed in ASD. First, studies on germ-free animals prove that the microbiota is important for maturation of the immune system and helps maintain immune homeostasis [93]. Plus, as previously mentioned, an immune challenge during pregnancy in the MIA model results in a dysbiosis in the offspring along with altered communication, social and repetitive behaviors and cortical defects similar to ASD [48,60,94]. Immune alterations similar to those seen in ASD patients were observed in MIA offspring, such as an increase in IL-6 and IL-17 pro-inflammatory cytokines, and higher proportion of Th17 cells [54,95]. Interestingly, in Hsiao et al. (2013) [54], a probiotic intervention with *B. fragilis NCTC 9343* was sufficient to restore normal IL-6 levels. More recently, a study by Kim et al. (2017) [95] demonstrated that a vancomycin treatment in MIA mothers during the whole gestational period prevented Th17 dysregulation in mothers and the appearance of behavioral and cortical alterations in offspring. The authors suspected that the vancomycin treatment induced a depletion of SFB which can induce T cell differentiation into Th17. Plus, they observed no MIA-induced behavioral phenotypes in mouse strains lacking SFB, and in consequence producing less Th17. Gavage of those mice with SFB was sufficient to restore MIA-induced phenotypes in the offspring. These data demonstrated that the presence of SFB in the gut, and consequent Th17 differentiation, were necessary to induce behavioral and cortical abnormalities in MIA offspring. 

It is interesting to note that immune dysregulations can also be observed in genetic or environmental models of ASD that are not related to an immune challenge. Plus, these dysregulations are often associated with bacterial modifications. In BTBR mice, an enhanced inflammatory response to LPS challenge has been observed, and basal colonic levels of TNF-α and IL-6 are elevated which correlates with some of the alterations in bacterial composition [52,96]. In models based on deletion or mutation of the Shank3 gene, systemic increase of IL-6 and IL-17 have been observed, as well as more GFAP-positive cells, which is a marker for astrogliosis. Interestingly, treatment with *L. reuteri* MM4-1A could lower IL-17 levels in this model [51,58]. In the MHFD model, an increase of IL-1β, IL-6 and TNF-α has been observed [55] as well as an increase in intestinal levels of IL-17 due to a higher proportion of innate lymphoid cells 3 (ILC3) cells in the intestinal lamina propria of the offspring. The authors treated pregnant mice with antibiotics to obtain offspring with depleted microbiota. These offspring were then transplanted with gut microbiota from either MHFD or control mice of the same age. They observed a higher proportion of ILC3 in offspring colonized with MHFD microbiota compared to offspring colonized with microbiota from controls. This result proved that the effect on ILC3 cells was dependent on the microbiota [64]. Finally, in the VPA model, various studies reported an increase of microglial density in various brain regions, and a LPS challenge induced overproduction of IL-6, IL-1β and TNF-α in the brain and in the spleen [56,97,98,99]. As previously mentioned, VPA mice and rats also have a disturbed microbiota with elevated butyrate production [61,63]. Butyrate is often considered a beneficial SCFA in gut-brain axis regulation, and has been found to enhance intestinal and blood-brain barrier (BBB) functions and promote anti-inflammatory responses [33]. However, de Theije et al. (2014) [56,63] proposed that the microbiota changes and the elevated butyrate levels they observed in the caecum of VPA mice could be associated to increased intestinal inflammation through modulation of the mucus composition. This lead would be interesting to pursue in order to gain a better understanding of the link between elevated butyrate and inflammation in this model.

Overall, those preclinical results show that immune challenges either during pregnancy or throughout life lead to ASD-like behaviors, and that this effect can be microbiota-dependent. Those observations and the fact that immune dysregulations are present in many ASD models, and often correlate with microbiota changes and altered behaviors, implies the existence of a microbiota-immune-brain relationship that could be part of the pathophysiology of ASD. However, most of the evidence of a gut microbiota-immune-brain axis in ASD is still based on preclinical research and there is a need for more clinical research on the subject.

### 2.3. Influence of the Gut Microbiota on Dysregulation of Tryptophan Metabolism in ASD

#### 2.3.1. Clinical Evidence

Trp cannot be produced by the body and only comes from dietary consumption. Dysregulations of the Trp metabolism in ASD have been described; however, their implication in the disorder is still unclear. Dietary Trp is the precursor for serotonin (5-HT) and kynurenine (KYN), whose pathways have been shown to be dysregulated in ASD [100]. KYN is derived from Trp via the indoleamine-2,3-dioxygenase (IDO), activated in presence of pro-inflammatory cytokines (IL-6 and IL-1) and TNF. KYN can then cross the BBB and be transformed into two derivatives, kynurenic acid (KA) or quinolinic acid (QA). KA is neuroprotective and reduces excitotoxicity via inhibition of the N-methyl-D-aspartate (NMDA) receptors, whereas QA is an agonist of those receptors and is thus neurotoxic [100,101]. Serum of ASD patients presents lower KA concentration [101,102], higher KYN/KA ratio and higher QA concentration [101]. Decrease of KA and increase of QA in the serum might reflect similar changes at the central level, thus leading to increased excitotoxicity, which may be involved in ASD pathophysiology.

The other main derivative of Trp is 5-HT. Ninety-five percent of 5-HT circulating in the body is produced by the gut enterochromaffin cells (ECs) through the action of the rate-limiting enzyme tryptophan hydroxylase 1 (TPH1) and of the aromatic acid decarboxylase (AADC). It plays a crucial role in regulation of GI functions. In the brain, 5-HT is produced via TPH2 and plays an important role in various brain functions such as mood, sleep or appetite regulation. Furthermore, both central and peripheral 5-HT play a role in pre- and postnatal neurodevelopment, thus their dysregulation has been hypothesized to be involved in ASD pathophysiology [103,104]. A few studies report central alterations of 5-HT in ASD patients. However, most of the evidence towards dysregulation of the 5-HT metabolism in ASD comes from the fact that increased blood levels of 5-HT have been widely observed in ASD patients since the 1960s, being found in more than 25% of them [104,105]. While this increase could be due to increased uptake by platelets or decreased breakdown, it could also be due to increased 5-HT release by ECs in the gut [104,106]. One study has found a small positive correlation between severity of GI symptoms and whole blood 5-HT levels in ASD patients [107]. Interestingly, Luna et al. (2017) [12] found decreased levels of Trp, and elevated levels of the 5-HT metabolite 5-HIAA in rectal tissue of ASD patients with GI dysfunction, and those modulations correlated with the increase or decrease of some bacterial species in the gut microbiota of those patients. To our knowledge, the effect of a probiotic intervention on the Trp pathway has not been investigated in ASD patients. However, it has been investigated in healthy subjects or patients with other pathologies. In a study by Kato-Kataoka et al. (2016) [108], daily intake of a fermented drink containing *L. casei* Shirota for 8 weeks prevented the elevation of plasmatic Trp of healthy subjects before a stressful examination period. In another study, after a long-term administration (105 days) of *L. reuteri* DSM-17938, adults suffering from functional constipation had lower plasmatic levels of 5-HT [109]. Finally, daily administration of probiotics for 8 weeks, either the *L. helveticus* R0052/*B. longum* R0175 mix or *L. plantarum* 299v, resulted in a decrease in seric KYN/Trp ratio and a decrease in seric KYN levels, respectively, in patients suffering from depressive disorders [110,111]. While these studies provide evidence that probiotic treatments can influence the Trp pathway, it is still unclear if the changes of microbiota in ASD are involved or not in the Trp alterations observed in ASD patients.

#### 2.3.2. Preclinical Evidence

Multiple preclinical studies have proved that the microbiota can influence Trp metabolism. Clarke et al. (2012) [112] have observed increased plasmatic Trp and a decreased plasmatic KYN/Trp ratio in male and female GF mice, the latter being restored by gut colonization with SPF microbiota. Plus, two separate studies found that GF mice had lower colonic levels of 5-HT and lower colonic expression of TPH1 mRNA, compared to SPF mice or mice colonized with microbiota from healthy human donors [113,114]. The study from Yano et al. (2015) [114] found an increased expression of the 5-HT transporter gene, SLC6A4, which they hypothesize to be a compensatory response to the deficit in 5-HT synthesis. Interestingly, colonization of GF mice at postnatal day 42 with spore-forming bacteria from either SPF mice or healthy human donors restored colonic and seric levels of 5-HT and normal TPH1 and SLC6A4 gene expression in the colon. The other study, from Reigstad et al. (2015) [113] found that in vitro stimulation of human-derived ECs with acetate or butyrate could induce TPH1 expression. Overall, those results show that certain types of bacteria from the gut microbiota and their metabolites can influence Trp metabolism along the 5-HT and KYN pathways.

Interestingly, BTBR, MIA, CMA and VPA mouse models of ASD all show impaired 5-HT metabolism. MIA mice present increased serum 5-HT [54], and CMA mice have increased 5-HT but decreased 5-HIAA in the ileum [50]. A decrease of intestinal 5-HT was also found in BTBR mice, as well as an increase in 5-HT/5-HIAA ratio [53]. In the VPA model, de Theije et al. (2014) [56] observed decreased 5-HT levels in the ileum associated with fewer ECs. The authors also observed alterations in 5-HT metabolism in the brain, such as a decrease of 5-HT and increase in 5-HIAA/5-HT ratio in the prefrontal cortex and amygdala. Plus, a recent study found that MIA mice presented increased expression of the 5-HT2A receptor in the frontal cortex [94]. However, it is still unclear if those central alterations are influenced by intestinal 5-HT.

As previously mentioned, microbiota alterations were described in all those models. Interestingly, in VPA, BTBR and MIA mice, the 5-HT alterations correlated with some of the observed microbiota changes [53,63,94].

Although most evidence towards an influence of the gut microbiota on alteration of neurotransmitter systems in ASD is focused around 5-HT, there has been sporadic evidence on involvement of the gut microbiota in other neurotransmitter systems related to ASD, particularly the GABAergic (gamma-aminobutyric acid) and glutamatergic systems. Clinical studies have found alterations in central or peripheric levels of GABA or glutamate, or altered expression of their receptors in the brain of ASD patients [31,115,116,117]. Some bacteria of the human gut microbiota are capable of producing GABA, which could be one of the ways that the microbiota impacts the gut-brain axis [118]. Interestingly, Kang et al. (2018) [30] found lower levels of GABA, as well as lower GABA/glutamate ratio in the feces of children with ASD. However, the authors found no correlation between these changes and the microbiota modulations observed in these patients. Another recent study looked at gene expression in the metagenome of ASD patients and found a decrease in genes related to GABA production [37]. More evidence comes from animal studies, as decreased expression of GABA receptors has been observed in the hippocampus of Shank3 KO mice. Interestingly, this alteration correlated with *L. reuteri* levels in the microbiota of those mice, and *L. reuteri* MM4-1A treatment partly restored those expression levels [58]. There is still little evidence of the implication of the microbiota in those GABA and glutamate alterations in ASD, but those first results provide a promising avenue to pursue.

In conclusion to this first part, it is now well-accepted that ASD patients have a disturbed microbiota, with altered metabolic activity. Increasing evidence shows that those disruptions can influence the immune system and Trp metabolism, both in the periphery and in the brain. Thus, the gut microbiota may have an influence on neurodevelopment and brain function during the life of ASD patients. These new findings have prompted many teams to test whether interventions on the gut microbiota could have beneficial effects on GI symptoms, brain function and behavior in ASD.

## 3. Clinical and Preclinical Interventions Targeting the Gut Microbiota

### 3.1. Probiotic Intervention Studies for ASD Symptoms 

Recently, a few interventional clinical studies and more interventional preclinical studies have been published, bringing evidence that modulation of the gut microbiota can influence ASD-related behaviors, as well as some elements explaining the underlying mechanisms of this effect. 

#### 3.1.1. Clinical Studies

As previously mentioned, one of the first studies to establish a link between gut microbiota dysbiosis and ASD was published in 2000 by Sandler et al. [13], who observed behavioral improvement of children with ASD during a vancomycin treatment, showing that modification of gut microbiota can induce changes in behavioral symptoms. However, those effects did not persist after the treatment, and a long-term antibiotic treatment is not feasible, thus, researchers have started to investigate the potential role of probiotic treatments in ASD. Multiple studies have reported effects of probiotic treatments on microbiota composition and GI symptoms in ASD children. Although many of these studies did not analyze the behavior of the children, or did not see any improvement after probiotic administration [26,119,120,121], other studies did report behavioral improvement [122,123,124,125]. In Shaaban et al. (2017) [123], 30 children with ASD were given a 3-month, daily treatment with a patented probiotic mixture (composed of strains of the species *L. acidophilus, L. rhamnosus* and *B. longum*) which induced an improvement in communication, sociability and cognitive awareness, characterized by a decrease in the ATEC (autism treatment evaluation checklist) score. In Liu et al. (2019) [124], *L. plantarum* PS128 was given to 36 children for 4 weeks in a placebo-controlled trial. The authors did not observe an improvement in behavioral scores using different diagnosis scales, but saw a decrease in anxiety behavior, hyperactivity and opposition/defiance behaviors. They propose that the effects of the treatment could have been stronger if it had been administered for a longer period of time. 

Overall, considering the variations in the probiotic choice, group size, duration of treatment and behavioral assessment tools, these results are not yet sufficient to establish a beneficial effect of probiotic interventions on behavior in ASD. However, the use of probiotics could be an interesting lead of treatment or preventive measures as suggested by numerous preclinical studies showing an effect of probiotics on behaviors related to ASD, which will be detailed later. Since each strain or even species could have a different influence on ASD symptoms, only an increased number of studies could allow to identify specific beneficial probiotic strains.

#### 3.1.2. Preclinical Studies

As previously mentioned, there are multiple murine models of ASD, genetic or environmental, that present altered behaviors relative to ASD. As many of those models also have impaired GI function and gut microbiota, as previously described, many research teams have wondered if modulating this microbiota composition using a probiotic treatment could improve the altered behaviors of those models. One of the first groups to publish such a study was Sarkis Mazmanian’s group, who tested the effect of *Bacteroides fragilis* NCTC 9343 in the MIA mouse model [54]. They observed an improvement in anxiety-like behavior, stereotyped behavior, communication and cognitive function. However, the probiotic treatment had no impact on social behavior. Interestingly, the authors identified one metabolite, 4-EPS, whose increased serum levels in MIA mice was restored to control values by the probiotic treatment. In parallel, a chronic systemic administration of 4-EPS to naïve mice induced an anxiety-like behavior. Since then, some other studies have tested probiotics in other mouse models of ASD. Buffington et al. (2016) [49], showed that *Lactobacillus reuteri* gut concentration was decreased in the MHFD mouse model, and that treatment with *L. reuteri* MM4-1A restored social behavior in those mice. Based on this observation, another team showed improvement of social and repetitive behavior in the Shank3 KO genetic model of ASD, following treatment with *L. reuteri* MM4-1A [58]. This has been later further explored by Sgritta et al. (2019) [62] who found that *L. reuteri* MM4-1A treatment improved social behavior in the VPA environmental model, the BTBR idiopathic model and the Shank3B KO genetic model. They also reported that administration of *L. reuteri* MM4-1A improved social behavior of GF mice, proving that this bacterium could act on its own. Interestingly, the same study also demonstrated that the effect of *L. reuteri* MM4-1A in Shank3B^-/-^ mice was dependent on the vagus nerve, as treatment with this bacterium was inefficient in vagotomized Shank3B^-/-^ mice. This work also brought a very thorough mechanistic explanation of the probiotic effect, showing that it was dependent on the presence of oxytocin receptors in the ventral tegmental area, which is involved in social interaction-induced neuronal plasticity [62].

These recent data provide good arguments on the potential effect of specific probiotic treatments on behavior in ASD patients, and on the mechanistic functioning of the microbiota-gut-brain axis in the context of this disorder. All these results obtained in rodents are a first step that will have to be confirmed in studies on ASD patients. In the coming years we should see the first studies published, owing to large-scale projects involving longitudinal surveys of children at risk for ASD and intervention trials with probiotics, e.g., the European-funded GEMMA project [126] or an American study testing the effects of an *L. reuteri* treatment, associated or not with an oxytocin nasal spray, on social behaviors in ASD patients [127].

### 3.2. FMT Studies

#### 3.2.1. Clinical Studies

To our knowledge, there are very few clinical studies exploring the impact of fecal microbiota transplantation (FMT), on ASD symptoms in patients. FMT is most commonly used as treatment of *C. difficile* infections, where it seems very efficient [128]. Besides, some studies have proven that FMT could have a therapeutic effect in patients with irritable bowel syndrome [129,130] and promising effects on insulin resistance and metabolic parameters in patients with metabolic syndrome, though this needs further investigation [131,132]. Some studies have also started to investigate the impact of FMT in various neurological disorders [133]. While those results are promising, it is worth noting that this procedure is not yet completely mastered and there is a need for more large-scale longitudinal studies. In 2017, Kang et al. published an open label study in which they performed FMT in 18 patients with ASD (7–16 years old) and comorbid GI symptoms [134]. Eighteen weeks after FMT, the team observed in all patients an amelioration of GI symptoms, an increase of microbiota diversity, assessed using Faith’s phylogenetic diversity index, and an amelioration of ASD-related behavioral symptoms, assessed according to clinical and parental based scales. The same team published a follow up study 2 years later, and the previously observed ameliorations had remained the same or even improved [135]. Those results offer a promising lead on the efficacy of FMT for amelioration of behavior and GI symptoms in ASD, but it needs to be further investigated in controlled studies with larger cohorts. 

#### 3.2.2. Preclinical Studies

The use of ASD mice models in preclinical studies is necessary for a more mechanistic understanding of the impact of FMT on microbiota, GI symptoms, other ASD-related markers and ASD-like behavior. Preclinical research has brought evidence of a potential therapeutic role of FMT in many neurological disorders, including ASD [133]. As previously mentioned, GF mice present impaired social interaction [49,66]. Interestingly, in Buffington et al. (2016) [49] colonization of GF mice at weaning with microbiota from normal mice normalized anxiety-like and social behaviors, while colonization with microbiota from MHFD mice did not. It is interesting to point out that the effects of the FMT did not appear when it was done at 8 weeks of age, highlighting the existence of a critical time window during which FMT in initially GF mice can impact behavior. In a recent study, Saunders et al. (2020) [94] transplanted gut microbiota from adult MIA mice or from control mice into 28-days-old control mice whose microbiota had been depleted by antibiotic treatment. The mice that received MIA microbiota showed impaired performances in the object recognition test compared to mice that received microbiota from controls. Overall, those results show that FMT from healthy mice can improve behavior in ASD models, whereas FMT from an ASD mouse model can induce behavioral deficits in healthy mice.

Recently, Sharon et al. (2019) [136] reported that transferring microbiota of children with ASD to GF mice could influence ASD-like behavior. GF mice were colonized at weaning with microbiota from ASD patients and from their typically developing (TD) siblings as control (humanization of mice). Their offspring (named ASD and TD, respectively) were tested for social interaction (three chamber sociability test), stereotyped behavior (marble burying test) and anxiety-like behavior (open-field). ASD mice presented decreased social interaction and increased stereotyped behavior compared to TD mice. Following this observation, the team observed a correlation between those behavioral differences and the higher or lower quantity of specific bacteria in ASD mice microbiota. They also observed alternate splicing for many genes in the prefrontal cortex, including a few that are known to be involved in some human cases of ASD or other neurodevelopmental syndromes. Finally, the team highlighted some metabolomic differences in colon content of ASD mice compared to TD mice, particularly a decrease in 5-aminovaleric acid and taurine, which could rescue some behavioral defects when administered to mice.

Another recent study performed FMT in the MIA mouse model, using pooled stools from three healthy human donors [60]. Mice received microbiota either directly after collection from the donors or after an in vitro culture step. Both FMT procedures reduced repetitive behavior assessed with the marble burying test and self-grooming analysis. Only the FMT with microbiota coming directly from the donors had an effect on anxiety-like behavior and none of the two FMT procedures had a significant impact on social behavior in the three-chamber social interaction test. 

FMT is a promising approach to improve behavior and GI symptoms in ASD patients. However, more clinical studies need to be done in order to reinforce this hypothesis. More preclinical studies are also necessary in order to gain more insight into the mechanisms by which FMT can induce systemic and neuronal changes leading to behavioral improvement.

## 4. Conclusions

Despite discrepancies between studies, the data presented in this review converge to conclude that ASD patients exhibit an abnormal microbiota composition, with altered activity. Whether these alterations are involved in the onset of ASD, or occur during the development of the disease, a growing body of research suggests that they may aggravate the behavioral symptoms and biological signs of ASD. 

**Figure 1:**
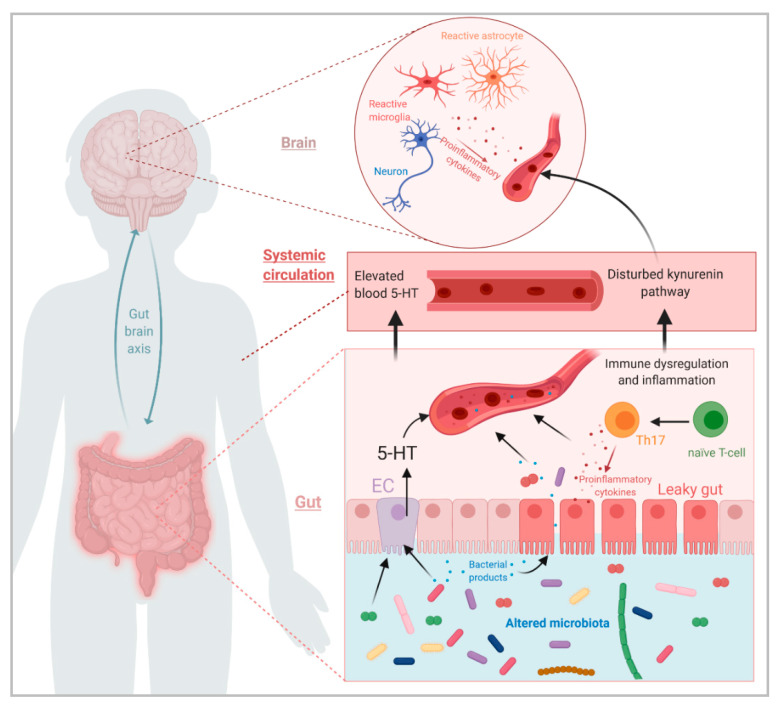
Summary schematic showing the potential impacts of a disturbed gut microbiota on various parameters in the gut, systemic circulation and brain in ASD, and how those parameters can be linked. (Figure created with BioRender.com).

This led to the use ASD animal models to try to elucidate the mechanisms underlying the involvement of the gut microbiota in this disorder. To date, these preclinical studies conducted in rodents have particularly shown alterations in the immune system and in the metabolism of Trp (summarized in Figure 1). In addition, recent studies bring evidence of a role of the gut microbiota, through its metabolites, in other neurological and physiological aspects that are disturbed in ASD patients, such as the GABAergic and glutamatergic transmission in the brain or respiratory mitochondrial activity [31,40,41].

Other factors could be associated with ASD and influenced by the gut microbiota. For example, it has been suggested that heavy metal imbalance could be involved in ASD, and studies show that heavy metals can influence gut-microbiota composition, that heavy metal imbalance is correlated with abundance of some bacterial genera, and that some bacterial species are capable of transforming heavy metals into more or less-toxic derivatives [137,138,139]. In a similar way, deficiencies in vitamin B levels, which have been found in ASD patients [140], could be linked to dysbiosis, considering the role of the gut microbiota in vitamin synthesis [141]. However, to our knowledge, there are still very few reports as of now of those interactions in the context of ASD, and this would be interesting to deepen the research on these subjects.

Recent clinical interventional studies, using probiotic treatments or FMT, have produced some promising results, supported by data from preclinical studies. Hopefully, by pursuing this back and forth between clinical and preclinical work, new evidence of the involvement of the intestinal microbiota in ASD are expected to be found, as well as new mechanisms underlying the action of the gut microbiota. Furthermore, since it is still unclear whether microbiota alterations appear as a consequence of ASD or are involved in its onset—there is also a need for longitudinal studies, in order to characterize when the microbiota comes into play in this disorder, and if it can be used as an early biomarker. This type of research will be implemented, in the coming years, for example by the European GEMMA project [126].

## Abbreviations

ASD	Autism spectrum disorder
BBB	Blood brain barrier
CMA	Cow’s milk allergy
EC	Enterochromaffin cells
4-EPS	4-Ethylphenylsulfate
FMT	Fecal microbiota transplantation
GABAergic	gamma-aminobutyric acid
GF	Germ free
GFAP	Glial fibrillary acidic protein
GI	Gastrointestinal
5-HT	Serotonin
IFN	Interferon
ILC3	Innate lymphoid cells
KA	Kynurenic acid
KYN	Kynurenin
LCL	Lymphoblastoid cell lines
LPS	Lipopolysaccharide
MHFD	Maternal high fat diet
MIA	Maternal immune activation
MPO	Myeloperoxidase
NMDA	N-methyl-D-aspartate
QA	Quinolinic acid
SCFAs	Short chain fatty acids
SFB	Segmented filamentous bacteria
SPF	Specific pathogen free
TD	Typically developing
Trp	Tryptophan
VPA	Valproic acid
WT	Wild-type

## Appendix A

**Table A1:** Microbiota modulations in genetic models of ASD.

Model	Sex	Sample	Method	Difference in Microbiota Compared to Controls	Ref.
Shank3^-/-^	F/M	Feces	16S rRNA seq RT-PCR	**α-diversity**: ↓ **β-diversity**: Modulated **Phylum level**: N.S **Class level**: ↓*Bacilli* **Order level**: ↓*Lactobacillales, Rhodospirillales, Rickettsiales and Turicibacteriales* **Family level**: *↑**Veillonellaceae*; ↓L*actobacillaceae, Bacteroidaceae, Acetobacteriaceae, mitochondria* and *Turicibacteriaceae;* *↑**Veillonellaceae* **Genus level**: ↓*Lactobacillus, Coprococcus, Bacteroides, Acetobacter, Turicibacter* and *Prevotella*; ↑*Veillonella* in males ↓ in females **Species level**: ↓*L. reuteri, L. brevis, L. ruminis* in both male and female; *↓**V. parvula* and *V. dispar* in females, *↑**V. dispar* in males	[58]
	N.S	Feces	16S rRNA seq	**No assessment of diversity** **Phylum level**: ↑*Actinobacteria* and *Firmicutes*; ↓ *Proteobacteria* Absence of *Verrucomicrobia*; Presence of *Deferribacteres, Chlamydiae* and *Tenericutes* **Class level**: N.S **Order level**: ↑ *Bifidobacteriales* and *Eggerthellales* **Family level**: N.S **Genus level**: ↑*Asaccharobacter, Eggerthella, Enterorhabdus* and *Paraeggerthella* **Species level**: ↑ *B. pseudolongum*, *Assacharobacter* WCA-131-CoC-2, *Eggerthella* YY7918 and *Enterorhabdus caecimuris*.	[51]
Shank3B^-/-^	M	Feces	16S rRNA seq	**α-diversity**: No changes **β-diversity**: Modulated Bacterial modulation were not detailed except for: **Species level**: ↓ *L. reuteri*	[62]
NL3^R451C^	M	Feces	ARISA * 16S rRNA seq	**α-diversity**: No changes **β-diversity**: Modulated at 3 weeks of age (not at 9 weeks) Bacterial modulations were only detailed at OTU level: **Species level (OTUs)**: *↑* OTUs from *Lachnospiraceae* family, ↓OTUs from *Candidate* phylum	[57]
BTBR	M	Feces and cecal content	16S rRNA seq	**α-diversity**: ↓ **β-diversity**: Modulated in cecal content only **Phylum level**: *↑ Bacteroidetes* in cecal content **Class level**: N.S **Order level**: N.S **Family level**: ↓ *Enterobacteriaceae* both cecal and fecal **Genus level**: N.S **Species level**: ↑ *A. Muciniphila, Lactobacillus* spp., *Roseburia* spp*., C. leptum, Prevotella* spp.; ↓ *Clostridium* cluster XI both cecal and fecal In cecal content only, ↑*Methanobrevibacter* spp.; ↓ *C. coccoides* and *Clostridium* cluster I In feces only, ↑ *C. coccoides and Clostridium cluster I;* *↓ Methanobrevibacter* spp.	[59]
	F/M	Feces	16S rRNA seq	**α-diversity**: No changes **β-diversity**: Modulated **Phylum level**: ↑ *Proteobacteria* and TM7 in female **Class level**: N.S **Order level**: N.S **Family level**: N.S **Genus level**: ↑*Bacteroides* and *Parabacteroides;* *↓Dehalobacterium* in both male and female. In females only, ↑*Prevotella, Coprobacillus, Sutterella, Akkermansia,* and unclassified genera of *Desulfovibrionaceae* and *Enterobacteriaceae* families*;* *↓ Oscillospira* and unclassified members of TM7 and *Rikenellaceae* families In males only, ↑ *Bacteroides, Parabacteroides, Lactobacillus, Coprobacillus* and unclassified genus of the *Helicobacteraceae* family; ↓ *Dehalobacterium, Ruminococcus* and *Desulfovibrio* **Species level**: N.S	[52]
	M	Cecal content	16S rRNA seq	**α-diversity**: ↓ **β-diversity**: Modulated **Phylum level**: ↑*Verrucomicrobia*, *Bacteroidetes;**↓ Firmicutes* and *Cyanobacteria* **Class level**: N.S **Order level**: N.S **Family level**: N.S **Genus level**: ↑*Akkermansia, Bacteroides, Bilophila, Enterorhabdus Intestinomonas* and S24-7; ↓ *Odoribacter, Parabacteroides, Rikenella, Blautia, Coprococcus, Bifidobacterium, Desulfovibrio, Lachnospiracae_Incertae Sedis* and RC9 gut group **Species level**: N.S	[53]
	M	Feces	16S rRNA seq	**α-diversity**: N.S **β-diversity**: Modulated Bacterial modulation were not detailed except for: **Species levels**: ↓ *L. reuteri*	[62]

N.S = Not specified. * ARISA = automated ribosomal intergenic spacer analysis

**Table A2:** Microbiota modulations in environmental models of ASD.

Model	Sex	Sample	Method	Difference in Microbiota Compared to Controls	Ref.
MIA	M/F	Feces	16S rRNA seq	**α-diversity**: No changes **β-diversity**: Modulated **Bacterial modulations were only detailed at OTU level**: ↑OTUs from the *Alphaproteobacteria* and *Bacili* classes, *Bacteroidales* order and *Prevotellaceae, Lachnospiraceae* and *Porphyromonadaceae* families ↓ OTUs from the *Actinobacteria* phylum, *Gammaproteobacteria, Mollicutes* and *Erysipelotrichi* classes and *Ruminococcaceae, Erysipelotrichaceae* and *Aligenaceae* families	[54]
	M	Cecal content	16S rRNA seq	**No assessment of diversity** **Phylum level**: N.S **Class level**: N.S **Order level**: N.S **Family level**: ↑ *Ruminococcaceae*, *Porphyromonadaceae, Aoerococcaceae* and *Erysipelotrichaceae* **Genus level**: ↑ *Candidatus* **Species level**: N.S	[94]
	N.S	Feces	16S rRNA seq	**α-diversity**: ↓ **β-diversity**: Modulated **Phylum level**: ↑*Bacteroidetes* and *Verrucomicrobia*; ↓ *Firmicutes* **Class level**: N.S **Order level**: N.S **Family level**: N.S **Genus level**: ↑*Prevotella, Prevotella_other, Akkermansia* and a genus of S24-7 family; ↓*Oscillospira, Ruminococcus, Bacteroides, Dehalobacterium, Desulfovibrio, Lactobacillus*, and members of the *Clostridiales* order and *Rikenellaceae, Lachnospiraceae* and *Ruminococcaceae* families. **Species level**: ↑ F16 and OTUs from the *Bacteroidales* order*, Clostridiaceae, Enterobacteriaceae* and *S24-7 familie* and *Akkermansia* and *Prevotella* genera ↓ OTUs from the *Clostridiales* order*, Ruminococcaceae* and *Rikenellaceae* families and *Ruminococcus, Bacteroides, Dehalobacterium, Desulfovibrio, Oscillospira* and *Odoribacter* genera	[60]
VPA	M/F	Feces	Total genomic DNA pyrosequencing	**α-diversity**: no changes **β-diversity**: no difference **Phylum level**: ↑ *Firmicutes;* *↓Bacteroidetes* **Class level**: N.S **Order level**: N.S **Family level**: N.S **Genus level**: ↑ Uncultured genus of *Erysipelotrichales,* uncultured genera of the *Bacteroidales* and *Desulfovibrionales* orders **Species level**: N.S	[63]
	M/F	Feces	16SrDNA seq	**α-diversity**: ↓ **β-diversity**: Modulated **Phylum level**: only in males ↑ *Bacteroidetes;* only in female, *↑ Actinobacteria* **Class level**: Only in males ↑*Bacteroida, Alphaproteobacteria;* *↓Coriobacteria* **Order level**: N.S **Family level**: ↑ *Eubacteriaceae, Rikenellaceae* and *Staphylococcaceae*; *↓ Enterobacteriaceae* **Genus level**: *↑ Anaerofustis, Proteus, Staphylococcus,* and *Allobaculum* Only in females ↑, *Bifidobacterium, Odoribacter* and *Candidatus* Arthromitus **Species level**: ↑ *Ruminococcus flavefaciens*, OTUs from the *Clostridiales* order and the *Ruminoccus* and S24-7 genera.	[61]
MHFD mice	M	Feces	16SrDNA seq	**α-diversity**: ↓ **β-diversity**: Modulated **No detail of the changes in bacterial taxa**	[49]
	N.S	Feces	16SrDNA seq	**α-diversity**: ↓ **β-diversity**: Modulated **Phylum level**: ↑ *Firmicutes, Verucomicrobia,* *↓Bacteroidetes* **Class level**: N.S **Order level**: N.S **Family level**: ↑*Peptostreptococcaceae* **Genus level**:↑*Streptococcus, Akkermansia* *↓ Lachnospiraceae_incertae_sedis* **Species level**: N.S	[55]

All studies used mice except for Liu et al. (2018) [58] who used rats. N.S = Not specified.
